# Spatio-temporal deep learning methods for motion estimation using 4D OCT image data

**DOI:** 10.1007/s11548-020-02178-z

**Published:** 2020-05-22

**Authors:** Marcel Bengs, Nils Gessert, Matthias Schlüter, Alexander Schlaefer

**Affiliations:** Institute of Medical Technology, Hamburg University of Technology, Hamburg, Germany

**Keywords:** 4D deep learning, Optical coherence tomography, Motion estimation, Regularization

## Abstract

**Purpose:**

Localizing structures and estimating the motion of a specific target region are common problems for navigation during surgical interventions. Optical coherence tomography (OCT) is an imaging modality with a high spatial and temporal resolution that has been used for intraoperative imaging and also for motion estimation, for example, in the context of ophthalmic surgery or cochleostomy. Recently, motion estimation between a template and a moving OCT image has been studied with deep learning methods to overcome the shortcomings of conventional, feature-based methods.

**Methods:**

We investigate whether using a temporal stream of OCT image volumes can improve deep learning-based motion estimation performance. For this purpose, we design and evaluate several 3D and 4D deep learning methods and we propose a new deep learning approach. Also, we propose a temporal regularization strategy at the model output.

**Results:**

Using a tissue dataset without additional markers, our deep learning methods using 4D data outperform previous approaches. The best performing 4D architecture achieves an correlation coefficient (aCC) of 98.58% compared to 85.0% of a previous 3D deep learning method. Also, our temporal regularization strategy at the output further improves 4D model performance to an aCC of 99.06%. In particular, our 4D method works well for larger motion and is robust toward image rotations and motion distortions.

**Conclusions:**

We propose 4D spatio-temporal deep learning for OCT-based motion estimation. On a tissue dataset, we find that using 4D information for the model input improves performance while maintaining reasonable inference times. Our regularization strategy demonstrates that additional temporal information is also beneficial at the model output.

## Introduction

Optical coherence tomography (OCT) is an image modality that is based on optical backscattering of light and allows for volumetric imaging with a high spatial and temporal resolution [21]. The imaging modality has been integrated into intraoperative microscopes [15] with applications to neurosurgery [8] or ophthalmic surgery [6]. Moreover, OCT has been used for monitoring laser cochleostomy [19].

While OCT offers a high spatial and temporal resolution, its field of view (FOV) is typically limited to a few millimeters or centimeters [14]. Therefore, during intraoperative imaging, the current region of interest (ROI) can be lost quickly due to tissue or surgical tool movement, which requires constant tracking of the ROI and corresponding adjustment of the FOV. Performing the adjustment manually can disrupt the surgical workflow which is why automated motion compensation would be desirable. In addition to that, some surgical procedures such as laser cochleostomy also require adjustment of a surgical tool in case patient motion occurs [28]. Due to the small scale of the cochlea structure, accurate adjustment is critical to avoid damaging surrounding tissue [3]. Both motion compensation for the adjustment of the OCT’s FOV and the adjustment of surgical tools require accurate motion estimation.

One approach is to use an external tracking system for motion estimation. For example, Vienola et al. used this approach with a scanning laser ophthalmoscope for motion estimation in the context of FOV adjustment [24]. Also, external tracking systems have been used in the context of cochleostomy [5, 7]. Alternatively, the OCT images can be used directly for motion estimation as OCT already offers a high spatial resolution. For example, Irsch et al. estimated the tissue surface distance from A-scans for axial FOV adjustment [13]. Also, Laves et al. used conventional features such as SIFT [18] and SURF [1] with 2D maximum intensity projects for motion estimation in the context of volume of interests stabilization with OCT [17]. Another approach for high-speed OCT tracking relied on phase correlation for fast motion estimation from OCT images [20]. These approaches rely on hand-crafted features which can be error-prone, and the overall motion estimation accuracy is often limited [16]. Therefore, deep learning methods have been proposed for motion estimation from OCT data. For example, Gessert et al. proposed using 3D convolutional neural networks (CNNs) for estimating a marker’s pose from single 3D OCT volumes [10]. For estimating the motion between two subsequent OCT scans, Laves et al. adopted a deep learning-based optical flow method [12] using 2.5D OCT projections [16]. Similarly, Gessert et al. proposed a deep learning approach for motion estimation where the parameters for a motion compensation system are directly learned from 3D OCT volumes by a deep learning model [9].

So far, deep learning-based motion estimation with OCT relied on an initial template volume and a moving image, following the concept of registration-based motion estimation, for example, using phase correlation [20]. This can be problematic if motion between the original template and the current state is very large as the overlap between the images becomes small. Modern OCT systems could overcome this problem by acquiring entire sequences of OCT volumes, following the motion trajectory, as very high acquisition rates have been achieved [25]. Therefore, more information can be made available between an initial state and the current state which could be useful for motion estimation. While deep learning approaches using two images could follow the trajectory with pair-wise comparisons, we hypothesize that processing an entire sequence of OCT volumes at once might provide more consistence and improved motion estimation performance.

**Fig. 1:**
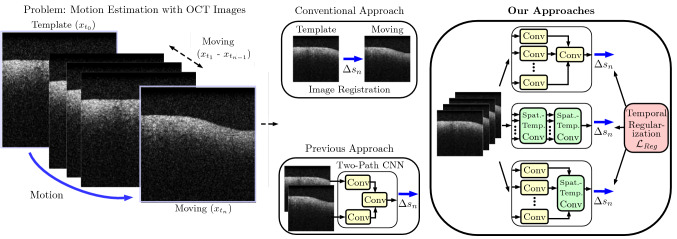
Our approach for motion estimation in comparison with previous methods. The approach is illustrated for 2D OCT images for simplicity. Note, we perform all experiments with 3D volumetric OCT images and thus 4D spatio-temporal data

In this paper, we compare several deep learning methods and investigate whether using 4D spatio-temporal OCT data can improve deep learning-based motion estimation performance, see Fig. 1. Using 4D data with deep learning methods is challenging in terms of architecture design due to the immense computational and memory requirements of high-dimensional data processing. In general, there are only few approaches that studied 4D deep learning. Examples include application to functional magnetic resonance imaging [2, 29] and computed tomography [4, 23]. This work focuses on studying the properties of deep learning-based motion estimation and the challenging problem of learning from high-dimensional 4D spatio-temporal data. First, we design a 4D convolutional neural network (CNN) that takes an entire sequence of volumes as the input. Second, we propose a mixed 3D–4D CNN architecture for more efficient processing that performs spatial 3D processing first, followed by full 4D processing. Third, we also make use of temporal information at the model output by introducing a regularization strategy that forces the model to predict motion states for previous time steps within the 4D sequence. For comparison, we consider a deep learning approach using a template and a moving volume as the input [9] which is common for motion estimation [16]. In contrast to previous deep learning approaches [9, 10], we do not use an additional marker and estimate motion for a tissue dataset. We evaluate our best performing method with respect to robustness toward image rotations and motion distortions. In summary, our contributions are threefold. First, we provide an extensive comparison of different deep learning architectures for estimating motion from high-dimensional 4D spatio-temporal data. Second, we propose a novel architecture that significantly outperforms previous deep learning methods. Third, we propose a novel regularization strategy, demonstrating that additional temporal information is also beneficial at the model output.

## Methods

### Experimental setup

**Fig. 2:**
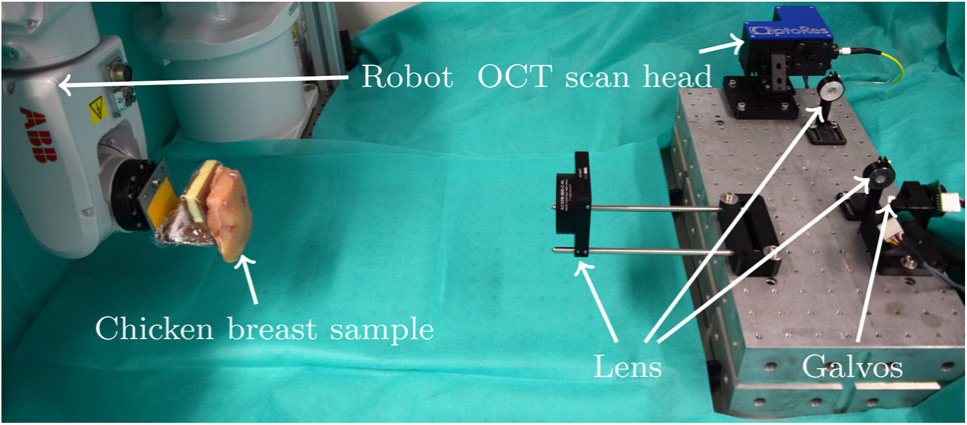
The experimental setup for data acquisition and annotation. The chicken breast sample is attached with needles to a holder of the robot. The OCT device itself is not shown

For evaluation of our motion estimation methods, we employ a setup which allows for automatic data acquisition and annotation, see Fig. 2. We use a commercially available swept-source OCT device (OMES, OptoRes) with a scan head, a second scanning stage with two mirror galvanometers, lenses for beam focusing and a robot (ABB IRB 120). The OCT device is able to acquire a single volume in 1.2 ms. A chicken breast sample is attached with needles to a holder of the robot. Our OCT setup allows for shifting the FOV without moving the scan head by using the second mirror galvanometers stage and by changing the pathlength of the reference arm. Two stepper motors control the mirrors of the second scanning stage, which shift the FOV in the lateral directions. A third stepper motor changes the pathlength of the reference arm to translate the FOV in the axial dimension. For evaluation of our methods, we consider volumes of size $32\times 32\times 32$ with a corresponding FOV of approximately $5\,\mathrm {mm}\times 5\mathrm {\,mm}\times 3.5\mathrm {\,mm}$.

### Data acquisition

We consider the task of motion estimation of a given ROI with respect to its initial position. To assess our methods on various tissue regions, we consider 40 randomly chosen ROIs of a chicken breast sample with the same size as the OCT’s FOV.

For motion estimation, only the relative movement between the FOV and ROI is relevant; hence, moving the ROI and using a steady FOV is equivalent to moving the FOV and using a steady ROI. This can be exploited for generation of both OCT and ground-truth labels. By keeping the ROI steady and moving the FOV by a defined shift in stepper motor space, we simulate relative ROI movement. At the same time, the defined shift provides a ground-truth motion as we can transform the shift in motor space to the actual motion in image space using a hand-eye calibration.

**Fig. 3:**
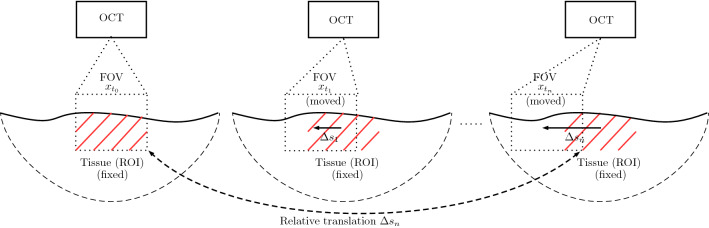
Our data acquisition strategy. For motion estimation only the relative movement is relevant; hence, we use a fixed ROI and move the FOV step-wise by ${\varDelta } s_{i}-{\varDelta } s_{i-1}$. This results in a sequence of OCT volumes $x_{t}$ with the corresponding relative translation ${\varDelta } s$ between the initial volume $x_{t_{0}}$ and the last volume $x_{t_{n}}$ of a sequence

Initially, the FOV completely overlaps with the target ROI. After acquiring an initial template image volume $x_{t_{0}}$ of the ROI, we use the stepper motors to translate the FOV by ${\varDelta } s_{t_{1}}$ such that the target ROI only partially overlaps with the FOV. Now, we acquire an image volume $x_{t_{1}}$ for the corresponding translation ${\varDelta } s_{t_{1}}$. This step can be repeated multiple times, resulting in a sequence of shifted volumes $x_{t_{i}}$ and known relative translations ${\varDelta } s_{t_{i}}$ between the initial ROI and a translated one. Note, each translation ${\varDelta } s_{t_{i}}$ is relative to the initial position of a ROI. The procedure is illustrated in Fig. 3.

In this way, we formulate a supervised learning problem where we try to learn the relative translation ${\varDelta } s_{t_{n}}$ of an ROI experiencing motion with respect to its initial position, given a sequence of volumes $x_{{t}}= \{x_{t_{0}},\ldots ,x_{t_{n}}\}$.

**Fig. 4:**
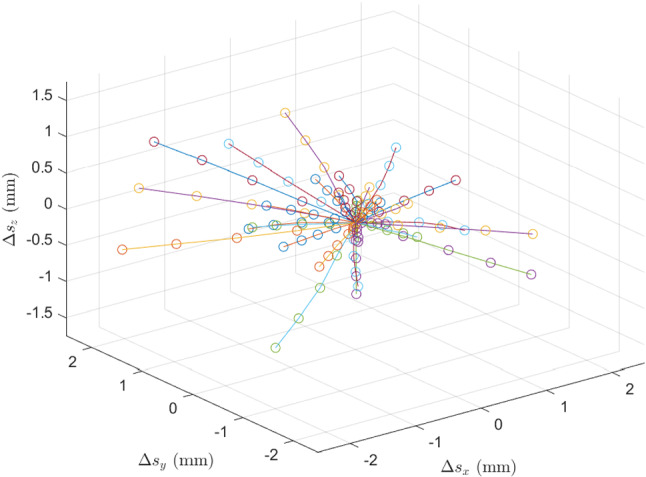
Shown are 30 example trajectories for the translations in the spatial dimensions, each trajectory consists of a sequence of five target shifts ${\varDelta } s_{i}$ (circle)

For generation of a single motion trajectory, we consider a sequence of five target translations, i.e., target motor shifts ${\varDelta } s_{t} = [{\varDelta } s_{t_{0}},{\varDelta } s_{t_{1}},{\varDelta } s_{t_{2}},{\varDelta } s_{t_{3}},{\varDelta } s_{t_{4}} ]$. To generate a smooth motion pattern, we randomly generate ${\varDelta } s_{t_{4}}$ and use spline interpolation between ${\varDelta } s_{t_{0}}=[0,0,0]$, ${\varDelta } s_{t_{4}}$ and a randomly generated connection point ${\varDelta } s_{c}$. We sample the intermediate target shifts ${\varDelta } s_{t_{1}},{\varDelta } s_{t_{2}},{\varDelta } s_{t_{3}}$ from the spline function. This results in various patterns where the FOV drifts away from the ROI. By using different distances between ${\varDelta } s_{0}$ and ${\varDelta } s_{4}$ we simulate different magnitudes of motions and obtain various different motor shift distances between subsequent volumes. Example trajectories are shown in Fig. 4. We use a simple calibration between galvo motor steps and image coordinates, to transform the shifts from stepper motor space to image space, resulting in a shift in millimeters.

For data acquisition, we use the three following steps. First, we use the robot for randomly choosing an ROI. Then, the initial state of the three motors corresponds to an FOV completely overlapping with the ROI. Second, we randomly generate a sequence of five target motor states, as described above, which shifts the FOV out of the ROI. Third, at each of the target motor states, an OCT volume is acquired.

Overall, for each ROI, we acquire OCT volumes of 200 motion patterns, where each movement consists of five target translations and five OCT volumes.

Moreover, we evaluate how the estimation performance is affected by relative rotations between volumes of a sequence. Note, our current scanning setup is designed for translational motion as rotation is difficult to perform using galvo mirrors. Therefore, we add rotations in a post-processing step, by rotating acquired volumes of a sequence $x_{{t}}$ around the axial axis. We define a maximal rotation $\alpha _{{max}}$ and transform each volume of a sequence with ${\widetilde{x}}_{{i}}=R(\alpha _{{i}})x_{{i}}$, while $\alpha _{i}=\frac{\alpha _{max}}{4}\cdot i,\,\,\forall i\in [0,4]$. Note, $R(\alpha _{{i}})$ is the rotation matrix for rotations around the depth axis. First, we consider rotations as noise that is applied to the image data. Second, we incorporate the rotation into our motion and adapt the ground truth with respect to the rotation.

Last, we also consider the effect of fast and irregular motion, such as high-frequency tremors that may cause distortion within an image. This effect is unlikely to occur with our current setup as our high acquisition frequency prevents common motion artifacts [27]. Nevertheless, we perform experiments with simulated motion artifacts due to relevance for slower OCT systems. We follow the findings of previous works [14, 26, 27] and consider motion distortions as lateral and axial shifts between B-scans of an OCT volume that has been acquired without motion distortions. In this way, we are able to augment our data with defined motion distortions in a post-processing step. To simulate different intensities of motion distortions, we introduce a factor $p_{dist}$ that defines the probability that a B-scan is shifted. Also, we compare shifting the B-scans one or two pixels randomly along the spatial dimensions.

### Deep learning models

**Fig. 5:**
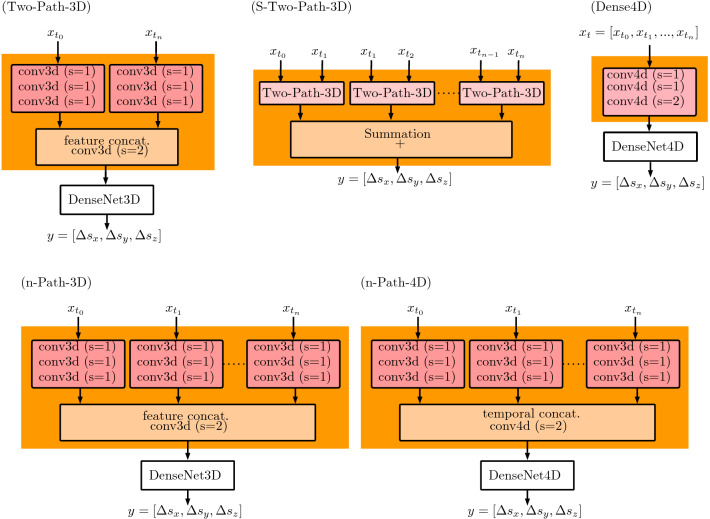
Our proposed network architectures. The networks receive volumes $x_{t_{i}}$ from a stream of volumes to predict the motion between the volumes $x_{t_{0}}$ and $x_{t_{n}}$. Note, for the multi-path architectures, the weights are shared across the paths

All our deep learning architectures consist of an initial processing block and a baseline block. For the baseline block, we adapt the idea of densely connected neural networks (densenet) [11]. Our baseline block consists of three densenet blocks connected by average pooling layers. Each densenet block consists of 2 layers with a growth rate of 10. After the final densenet block, we use a global average pooling layer (GAP) for connecting the three-dimensional linear regression output layer. Note, the output *y* of the architecture is the relative translation between volume $x_{t_{0}}$ and $x_{t_{n}}$ in all spatial directions. Using this baseline block, we evaluate five different initial processing concepts for motion estimation based on 4D OCT data, shown in Fig. 5.

First, we follow the idea of a two-path architecture for OCT-based motion estimation [9]. This architecture individually processes two OCT volumes up to a concatenation point by a two-path CNN with shared weights. At the concatenation point, the outputs of the two paths are stacked into the channel dimension and subsequently processed jointly by a 3D CNN architecture. In this work, we use three CNN layers for the initial two-path part and our densenet baseline block with 3D convolutions (DensNet3D) for processing after the concatenation point. In the first instance, we only consider the initial volume $x_{t_{0}}$ and the last volume $x_{t_{n}}$ of a sequence to estimate the relative translation. We refer to this architecture as Two-Path-3D.

Second, we use Two-Path-3D and consider predicting the relative translation between the initial and last volume, based on the sum of the relative translations between two subsequent volumes of a sequence. In this way, the network obtains information from the entire sequence. The network receives the input pairs [$x_{t_{0}}$, $x_{t_{1}}$], [$x_{t_{1}}$, $x_{t_{2}}$], [$x_{t_{1}}$, $x_{t_{2}}$], [$x_{t_{2}}$, $x_{t_{3}}$], [$x_{t_{3}}$, $x_{t_{4}}$], and the estimations are added to obtain the final network prediction *y*. Note, we train our network end-to-end based on the relative translation between the initial and the last volume and the network prediction *y*. We refer to this architecture as S-Two-Path-3D.

Third, we extent the idea of a two-path architecture to processing of an entire sequence of volumes, instead of using only two volumes as the networks input. For this purpose, we extend the two-path architecture to a multi-path architecture, while the number of paths is equal to number of volumes used. Note, similar to the two-path CNN, the multi-path layers consists of three layers with shared weights, followed by our densenet baseline block with 3D convolutions (DensNet3D). We refer to this architecture as Five-Path-3D.

Fourth, we use a 4D convolutional neural network, which employs 4D spatio-temporal convolutions and hence jointly learns features from the temporal and spatial dimensions. The input of this network is four dimensional, (three spatial and one temporal dimension) using a sequence of volumes. This method consists of an initial convolutional part with three layers, followed by our densenet block using 4D convolutions throughout the entire network. We refer to this architecture as Dense4D.

**Table 1:** Comparison of the different models for motion estimation. Our errors refer to the translation ${\varDelta } s$ between the template and the last volume of a motion sequence. Errors are given in mm

	MAE ${\varDelta } s_{x}$	MAE ${\varDelta } s_{y}$	MAE ${\varDelta } s_{z}$	rMAE	aCC (%)
Two-Path-3D	$0.45\pm 0.52$	$0.42\pm 0.52$	$0.18 \pm 0.15$	$0.34\pm 0.39$	85.47
S-Two-Path-3D	$0.20\pm 0.21$	$0.15\pm 0.16$	$0.13 \pm 0.12$	$0.16\pm 0.17$	97.70
Five-Path-3D	$0.35\pm 0.45$	$0.18\pm 0.25$	$0.11\pm 0.09$	$0.21\pm 0.26$	93.39
Dense4D	$0.22 \pm 0.21$	$0.20 \pm 0.24$	$0.13 \pm 0.11$	$0.19 \pm 0.19$	96.86
**Five-Path-4D**	$\mathbf {0.16}\pm \mathbf {0.18}$	$\mathbf {0.13}\pm \mathbf {0.15}$	$\mathbf {0.10} \pm \mathbf {0.09}$	$\mathbf {0.13}\pm \mathbf {0.14}$	$\mathbf {98.58}$

Bold highlights the best performing method/results

Fifth, we combine the idea of 4D spatio-temporal CNNs and multi-path architectures. At first, we split the input sequence and use a multi-path 3D CNN to individually process each volume of the sequence. However, instead of concatenating the volumes along the feature dimension at the output of the multi-path CNN, we reassemble the temporal dimension by concatenating the outputs into a temporal dimension. Then, we employ our DenseNet4D baseline block. We refer to this architecture as Five-Path-4D.

### Training and evaluation

We train our models to estimate the relative motion of an ROI using OCT volumes. Hence, we minimize the mean squared error (MSE) loss function between the defined target motions ${\varDelta } s_{t_{n}}$ and our predicted motions $y_{t_{n}}$.

1 $$\begin{aligned} {\mathcal {L}}=\frac{1}{N}\sum _{j=1}^{N}\left\| {\varDelta } s_{t_{n}}^{\{j\}}-y_{{t_{n}}}^{\{j\}}\right\| ^{2} \end{aligned}$$

Our goal is to estimate the relative motion between an initial volume $x_{t_{0}}$ and a final volume $x_{t_{n}}$, corresponding to the target shift ${\varDelta } s_{t_{n}}$. Given the nature of our acquisition setup, the intermediate shifts ${\varDelta } s_{t_{i}}$ are also available. As these additional shifts represent additional motion information, we hypothesize that they could improve model training by enforcing more consistent estimates and thus regularize the problem.

We incorporate the additional motion information by forcing our models to also predict the relative shifts of previous volumes $x_{{t_{n-1}}}$ and $x_{{t_{n-2}}}$. Thus, we also consider the relative translations ${\varDelta } s_{t_{n-1}}$ and ${\varDelta } s_{t_{n-2}}$ and we extent the network output by also predicting $y_{{t_{n-1}}}$ and $y_{{t_{n-2}}}$. Note, the additional output $y_{{t_{n-1}}}$ and $y_{{t_{n-2}}}$ is only considered during training and not required for application.

For optimization, we propose and evaluate the following loss function and introduce parameters $w_{n-1}, w_{n-2}\in [0,1]$ for weighting of the additional temporal information, introduced as a regularization term.

2 $$\begin{aligned} {\mathcal {L}}= & {} \frac{1}{N}\sum _{j=1}^{N}\left\| {\varDelta } s_{t_{n}}^{\{j\}}-y_{{t_{n}}}^{\{j\}}\right\| ^{2}+w_{n-1}\left\| {\varDelta } s_{t_{n-1}}^{\{j\}}-y_{{t_{n-1}}}^{\{j\}}\right\| ^{2} \nonumber \\&+w_{n-2}\left\| {\varDelta } s_{t_{n-2}}^{\{j\}}-y_{{t_{n-2}}}^{\{j\}}\right\| ^{2} \end{aligned}$$

We train all our models for 150 epochs, using Adam for optimization with a batch size of 50. To evaluate our models on previously unseen tissue regions, we randomly choose five independent ROIs for testing and validating each. For training, we use the remaining 30 ROIs.

## Results

First, we compare the different methods and report the mean absolute error (MAE), the relative mean absolute error (rMAE) and average correlation coefficient (aCC) for our experiments in Table 1. The MAE is given in mm based on the calibration between galvo motor steps and image coordinates. The rMAE is calculated by dividing the MAE by targets’ standard deviation. We state the number of parameters and inference times for all models, see Table 2. For all experiments, we test our results for significant differences in the median of the rMAE using Wilcoxon signed-rank test with $\alpha = 0.05$ significance level. Overall, using a sequence of volumes improves performance significantly and Five-Path-4D performs best with a high aCC of 98.58%. Comparing Five-Path-4D to Two-Path-3D, the rMAE is reduced by a factor of approximately 2.6. Moreover, employing the two-path architecture on subsequent volumes and adding the estimations (S-Two-Path-3D) perform significantly better than directly using the initial and the last volume (Two-Path-3D) of a motion sequence.

**Table 2:** Number of parameters and inference times for all models

	Number of parameters	Inf. time (ms)
Two-Path-3D	143913	$3.74\pm 0.52$
S-Two-Path-3D	143913	$5.84\pm 0.32$
Five-Path-3D	208713	$5.23\pm 0.27$
Dense4D	270283	$9.78\pm 0.74$
Five-Path-4D	258323	$9.34\pm 0.67$

**Fig. 6:**
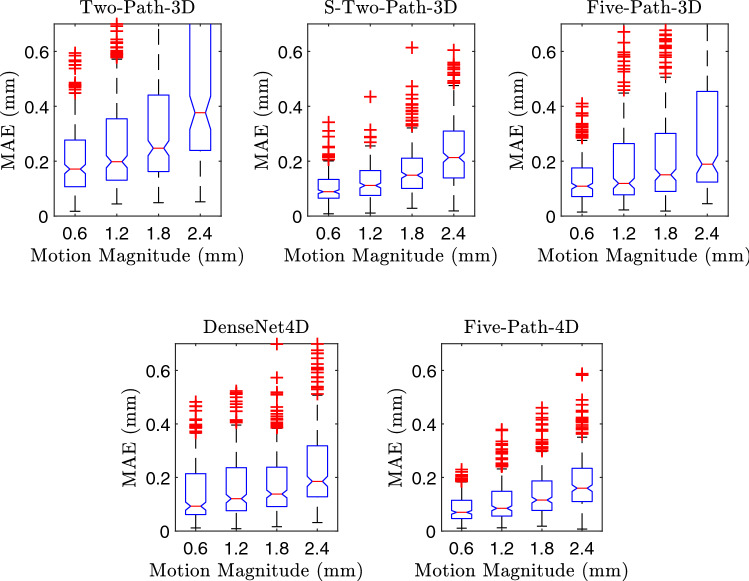
MAE for increasing motion magnitudes. Results are shown for four motion groups, covering increasing magnitudes of motion

Second, we extent the comparison of our models and present the MAE over different motion magnitudes, shown in Fig. 6. The error increases with an increasing magnitude of the motion for all models. Comparing the different models shows that the error increases only slightly for Five-Path-4D, compared to the other models.

**Table 3:** Evaluation of the performance for different rotation angels during motion. We evaluate the rotation as noise or as part of the motion, where the ground truth ${\varDelta } s$ is rotated accordingly. The rotation angle $\alpha _{max}$ refers to the relative rotation between the initial template volume and the last volume of a sequence. Results are shown for the architecture Five-Path-4D. The errors refer to the translation ${\varDelta } s$ between the template and the last volume of a motion sequence and are given in mm

	$\alpha _{max}$	MAE ${\varDelta } s_{x}$	MAE ${\varDelta } s_{y}$	MAE ${\varDelta } s_{z}$	rMAE	aCC (%)
Noise	$2^\circ$	$0.17 \pm 0.18$	$0.13 \pm 0.15$	$0.10 \pm 0.09$	$0.13\pm 0.14$	98.56
	$5^\circ$	$0.19 \pm 0.18$	$0.15 \pm 0.15$	$0.09\pm 0.09$	$0.14 \pm 0.14$	98.44
	$10^\circ$	$0.23 \pm 0.19$	$0.16\pm 0.16$	$0.10\pm 0.09$	$0.17\pm 0.14$	97.95
	$20^\circ$	$0.34 \pm 0.25$	$0.23\pm 0.20$	$0.10 \pm 0.09$	$0.22 \pm 0.18$	96.04
Motion	$2^\circ$	$0.16\pm 0.18$	$0.13\pm 0.15$	$0.09\pm 0.09$	$0.13\pm 0.14$	98.60
	$5^\circ$	$0.16\pm 0.18$	$0.14\pm 0.15$	$0.10\pm 0.09$	$0.14\pm 0.14$	98.55
	$10^\circ$	$0.17\pm 0.18$	$0.16\pm 0.15$	$0.10\pm 0.09$	$0.15\pm 0.14$	98.35
	$20^\circ$	$0.19\pm 0.20$	$0.23\pm 0.19$	$0.10\pm 0.09$	$0.18\pm 0.16$	97.48

Third, Table 3 shows how rotations affect the performance for our best performing model Five-Path-4D during evaluation. First, we consider rotations as noise during motion and do not transform the target shifts. Second, we consider rotations as part of the motion and transform the target shifts accordingly. For small rotation angels $\alpha _{max}<5^{\circ }$, performance is robust and hardly reduced. For larger rotations angels $\alpha _{max}>5^{\circ }$, lateral estimation performance is affected when rotations are considered as noise, while performance remains similar when rotations are considered as part of the motion.

Fourth, Table 4 demonstrates how motion distortions affect performance. We evaluate different magnitudes of motion distortions. The results show that performance is hardly reduced when only few motion distortions are present ($p_{dist}<10\%$). However, as we increase the amount of motion distortions, performance is notably affected, yet, performance is recovered when distortions are also considered during training.

Fifth, we address the temporal regularization strategy, see Table 5 for our best performing model Five-Path-4D. We report performance metrics for various weighting factors $w_{n-1}$ and $w_{n-2}$. Our results demonstrate that using the regularization strategy improves performance. Fine-tuning the weights improves performance significantly with a high aCC of $99.06\%$ for a weighting of $w_{n-1}=0.75$ and $w_{n-2}=0.75$.

**Table 4:** Results for Five-Path-4D when motion distortions are applied during evaluation; $p_{dist}$ refers to the probability that a B-scan is shifted. We evaluate shifting the B-scans one (E-1) or two pixels (E-2) during evaluation. Also, we consider motion distortions of two pixels during training and evaluation (T/E-2). Our errors refer to the translation ${\varDelta } s$ between the template and the last volume of a motion sequence. Errors are given in mm

Type	$p_{dist}$(%)	MAE ${\varDelta } s_{x}$	MAE ${\varDelta } s_{y}$	MAE ${\varDelta } s_{z}$	rMAE	aCC (%)
E-1	50	$0.31\pm 0.33$	$0.29\pm 0.29$	$0.14\pm 0.11$	$0.25\pm 0.24$	94.41
E-1	25	$0.20\pm 0.22$	$0.20\pm 0.20$	$0.11\pm 0.10$	$0.17\pm 0.17$	97.37
E-1	10	$0.16\pm 0.18$	$0.16\pm 0.17$	$0.10\pm 0.09$	$0.14\pm 0.15$	98.25
E-2	50	$0.33\pm 0.35$	$0.28\pm 0.28$	$0.14\pm 0.12$	$0.25\pm 0.24$	94.27
E-2	25	$0.20\pm 0.21$	$0.20\pm 0.21$	$0.12\pm 0.10$	$0.17\pm 0.17$	97.39
E-2	10	$0.17\pm 0.18$	$0.15\pm 0.16$	$0.10\pm 0.09$	$0.14\pm 0.14$	98.27
T/E-2	50	$0.18\pm 0.21$	$0.15\pm 0.15$	$0.10\pm 0.08$	$0.14\pm 0.15$	97.97

**Table 5:** Evaluation of the temporal loss regularization using different weighing factors $w_{n-1}$, $w_{n-2}$. Results are shown for the architecture Five-Path-4D with respect to predicting the motion ${\varDelta } s$ between the template and the last volume of a sequence. Errors are given in mm

$w_{n-1}$	$w_{n-2}$	MAE ${\varDelta } s_{x}$	MAE ${\varDelta } s_{x}$	MAE ${\varDelta } s_{x}$	rMAE	aCC (%)
0	0	$0.16\pm 0.18$	$0.13\pm 0.15$	$0.10 \pm 0.09$	$0.13\pm 0.14$	98.58
1	0	$0.15 \pm 0.22$	$0.12\pm 0.13$	$0.11\pm 0.10$	$0.13\pm 0.15$	98.15
0.75	0	$0.14 \pm 0.13$	$0.11\pm 0.10$	$0.13\pm 0.10$	$0.14 \pm 0.11$	98.90
0.5	0	$0.10 \pm 0.09$	$0.14\pm 0.11$	$0.10\pm 0.08$	$0.12\pm 0.10$	99.02
0.25	0	$0.11\pm 0.11$	$0.14\pm 0.13$	$0.11 \pm 0.09$	$0.12\pm 0.11$	98.92
1	1	$0.11\pm 0.10$	$0.19\pm 0.17$	$0.10\pm 0.09$	$0.14 \pm 0.12$	98.71
**0.75**	**0.75**	$\mathbf {0.09}\pm \mathbf {0.09}$	$\mathbf {0.11}\pm \mathbf {0.10}$	$\mathbf {0.10}\pm \mathbf {0.08}$	$\mathbf {0.10}\pm \mathbf {0.09}$	$\mathbf {99.06}$
0.75	0.5	$0.12\pm 0.10$	$0.10 \pm 0.11$	$0.10\pm 0.08$	$0.11\pm 0.10$	99.03

Bold highlights the best performing method/results

## Discussion

Motion estimation is a relevant problem for intraoperative OCT applications, for example in the context of motion compensation [13] and surgical tool navigation [28]. While previous approaches for motion estimation relied on a template and moving images, we learn a motion vector from an entire sequences of OCT volumes. This leads to the challenging problem of 4D spatio-temporal deep learning.

We design three new CNN models that address 4D spatio-temporal processing in different ways. While Five-Path-3D is an immediate extension of the previous two-path approach [9], our Five-Path-4D and Dense4D models perform full 4D data processing. For a fair comparison, we also consider pair-wise motion estimation along the sequence using Two-Path-3D, aggregated to a final estimate. Our results in Table 1 show that the two-path method using only the start and the end volume performs worse than the other methods. This demonstrates that there is not enough information for motion estimation or the motion is too large.

For using a full sequence of volumes, the Five-Path-3D CNN performs significantly worse than the other deep learning approaches. This indicates that stacking multiple volumes in the models feature channel dimension is not optimal for temporal processing. This has also been observed for spatio-temporal problems in the natural image domain [22]. This is also supported by pair-wise processing with S-Two-Path-3D which shows a significantly higher performance than the feature stacking approach and a higher performance than Dense4D. Our proposed 4D architecture outperforms all other approaches, including the previous deep learning concepts using two volumes [9, 16] and pair-wise processing. Thus, we demonstrate the effective use of full 4D spatio-temporal information with a new deep learning model.

Next, we also consider the effect of different motor shift distances for our problem. Note, faster movements lead to larger distance between subsequent volumes of a sequence and to reduced overlap, making motion estimation harder as there are fewer features for finding correspondence. The results in Fig. 6 show the performance for different distances between volumes. As expected, we observe a steady increase with larger distances for all models. For the approaches using just two volumes, the increase is substantial, while it remains moderate for the 4D spatio-temporal models. Thus, 4D data are also beneficial for various magnitudes of motion to be estimated, and we demonstrate that the models effectively deal with different spatial distances between time steps.

Moreover, Table 3 shows how rotations affect performance for our best performing method when applied during evaluation. When rotations are considered as noise, only for large rotations $\alpha _{max}>5^{\circ }$ performance is notably reduced. However, when rotations are considered as part of the motion, performance remains similar even for larger rotations. As rotations were not present in the training data, the results indicate that our models are robust with respect to rotations.

Furthermore, we consider the problem of potential motion artifacts. The OCT device we employ is able to acquire an OCT volume in 1.2 ms. According to Zawadzki et al., motion artifacts are not present for volume acquisition speeds below 100 ms [27]. However, to ensure that our methods are applicable to slower OCT devices as well, we consider the effect of fast and irregular motion that may cause image distortions. We consider motion distortions as lateral or axial shifts between B-scans of an OCT volume, similar to previous works [14, 26, 27]. The results in Table 4 demonstrate that motion distortions applied only during evaluation can affect performance. This highlights the importance of fast volumetric imaging when 4D data are used for motion estimation. However, when motion artifacts are also considered during training, performance can be recovered. These results indicate that using deep learning with 4D data is a viable approach, even if data are affected by fast and irregular motion distortions.

As temporal information appears to be beneficial at the model input, we also consider usage at the model output. Here, we introduce a regularization strategy which forces the model to learn consecutive motion steps. We also introduce weighting factors for fine-tuning of our approach. Our results in Table 5 demonstrate that the regularization method appears to be effective. While a weighting equal to one does not lead to an immediate performance improvement, using a weighing of $w_{n-1}=0.75$, $w_{n-2}=0.75$ improves performance notably up to an aCC of 99.06 %. As a result, providing more information on the trajectory during training appears to be helpful for 4D motion estimation.

While our 4D deep learning methods significantly improve performance, their more costly 4D convolution operations also affect inference times which is important for application when real-time processing is required. Inference times in comparison with model size are shown in Table 2. While Five-Path-4D significantly outperforms S-Two-Path-3D in terms of motion estimation performance, S-Two-Path-3D allows for faster predictions. Thus, there is a trade-off between performance and inference time for the different architectures. However, with an inference time of 107 Hz, our 4D deep learning methods are already a viable approach for real-time motion estimation which could be improved in the future by using more powerful hardware or additional low-level software optimization.

## Conclusion

We investigate deep learning methods for motion estimation using 4D spatio-temporal OCT data. We design and evaluate several 4D deep learning methods and compare them to previous approaches using a template and a moving volume. We demonstrate that our novel 3D–4D deep learning method significantly improves estimation performance on a tissue data set, compared with the previous deep learning approach of using two volumes. We observe that large motion is handled well by the 4D deep learning methods. Also, we demonstrate the effectiveness of using additional temporal information at the network’s output by introducing a regularization strategy that forces the 4D model to learn an extended motion pattern. These results should be considered for future applications such as motion compensation or the adjustment of surgical tools during interventions. Also, our 4D spatio-temporal methods could be extended to other problems such as ultrasound-based motion estimation.
